# Relevance of the Extraction Stage on the Anti-Inflammatory Action of Fucoidans

**DOI:** 10.3390/pharmaceutics15030808

**Published:** 2023-03-01

**Authors:** Noelia Flórez-Fernández, Carlos Vaamonde-García, Maria Dolores Torres, Manuela Buján, Alexandra Muíños, Antonio Muiños, María J. Lamas-Vázquez, Rosa Meijide-Faílde, Francisco J. Blanco, Herminia Domínguez

**Affiliations:** 1 CINBIO, Departamento de Ingeniería Química, Campus Ourense, Universidade de Vigo, 32004 Ourense, Spain; 2 Grupo de Investigación de Reumatología y Salud (GIR-S), Departamento de Biología, Facultad de Ciencias, CICA-Centro Interdisciplinar de Química y Biología, INIBIC-Sergas, Universidade da Coruña, Campus da Zapateira, 15011 A Coruña, Spain; 3 Portomuíños, Polígono Industrial, Rúa Acebedo, Parcela 14, Cerceda, 15185 A Coruña, Spain; 4 Grupo de Terapia Celular y Medicina Regenerativa, Universidade da Coruña, CICA-Centro Interdisciplinar de Química y Biología, Complexo Hospitalario Universitario A Coruña, Campus Oza, 15006 A Coruña, Spain; 5 Grupo de Investigación de Reumatología y Salud (GIR-S), Departamento de Fisioterapia, Medicina y Ciencias Biomédicas, Facultad de Fisioterapia, CICA-Centro Interdisciplinar de Química y Biología, INIBIC-Sergas, Universidade da Coruña, Campus de Oza, 15006 A Coruña, Spain

**Keywords:** brown seaweed, sulphated polysaccharides, extraction, inflammation

## Abstract

The anti-inflammatory action of fucoidans is well known, based on both in vitro and some in vivo studies. The other biological properties of these compounds, their lack of toxicity, and the possibility of obtaining them from a widely distributed and renewable source, makes them attractive novel bioactives. However, fucoidans’ heterogeneity and variability in composition, structure, and properties depending on seaweed species, biotic and abiotic factors and processing conditions, especially during extraction and purification stages, make it difficult for standardization. A review of the available technologies, including those based on intensification strategies, and their influence on fucoidan composition, structure, and anti-inflammatory potential of crude extracts and fractions is presented.

## 1. Introduction

Fucoidans are heteropolysaccharides found in brown seaweeds. The variety of biological properties [1,2,3], their safety [4,5], and the possibility of obtaining them from cheap and renewable sources, makes them attractive bioactives for the development of novel drugs [6].

The biological activities of fucoidans depend on their composition (monosaccharides, sulfation degree, and position), structure (glycosidic linkages, molecular weight, branching, substitution degree, etc.), as well as the route of administration [7]. The seaweed source, species, environmental and collecting area, biotic and abiotic characteristics [8,9], as well as the processing conditions strongly affect the fucoidan composition and structure. These different features determine the biological properties that have been reported, including anticoagulant, antioxidant [10,11], antitumor [12,13], antiviral [14,15], anti-inflammatory [16,17,18,19], and immunomodulatory [5,7] properties.

The anti-inflammatory action of fucoidans has been related with the traditional medicine uses of some seaweeds, such as *Sargassum* sp. [20,21]. Fucoidans can inhibit inflammatory processes by different pathways and have also demonstrated inhibition of these processes in vivo [22]. Recently, they have been proposed for use as a potential anti-inflammatory drug [23,24]. Their safety has been confirmed by toxicity tests [24], and did not affect the viability of cells, including RAW264.7 cells [25,26,27,28], THP-1 macrophages [22,29], rabbit articular chondrocytes [30], colorectal cancer cells DLD-1 and SW480 [31], HaCaT, and Hs68 cells [26].

Recent works have reviewed the sources, chemistry, and activities [32,33,34], anti-inflammatory properties, and the molecular mechanisms [7,35,36]. Most studies have been performed with commercial crude fucoidans, but also with purified fractions. The major characteristics limiting the practical applications are the structural heterogeneity and the high molecular mass of polysaccharides, which could limit permeability through cell membranes. Therefore, the influence of the extraction technologies on the structural features deserves further studies.

The present review aims to present an overview of the anti-inflammatory potential of fucoidans, the major mechanisms of action, the relevance of the physicochemical features, and the importance of the processing conditions. A survey on the effect of different extraction/depolymerization technologies on these properties is shown. Particular emphasis is given to explore emerging extraction techniques, which offer environmental and operational advantages, for fucoidan extraction and depolymerization.

## 2. Fucoidans

These heterogeneous sulphated polysaccharides that are exclusive to brown seaweeds contain α-1,3-linked or α-1,4-linked L-fucose and other monosaccharides, such as galactose, mannose, xylose, arabinose, glucose uronic acids, and acetyl groups [6]. Fucoidans represent 5–25% of dry macroalgal biomass [37,38].

Different backbone structures of fucoidan have been described, with α-(1→2)-, α-(1→3)-, and/or α-(1→4)-glycosidic bonds [39]. In *Fucus* sp., fucoidans are formed by (1→3)- and (1→4)-linked α-L-Fucp residues, sulfated at C-2 and/or C-3 and/or occasionally at the C-4 position whereas in *Undaria pinnatifida* and some *Sargassum* species, the fucoidans consist of alternating (1→3)- or (1→4)-linked α-L-fucose and β-d-galactopyranose residues, usually sulfated linked at the C-2 position. Information on the chemical and structural characteristics can be found in a number of comprehensive reviews [2,3,6,9].

According to their composition, fucoidans have been classified into different types [35]. F-fucoidans, the most studied type and found in most commercial products, contain sulfated fucose as the predominant component [40]. The G-fucoidan type also contains galactose in important proportions. Other minor types are fucoidans containing uronic acids and sulfated xylofucans [34]. The G-fucoidans are heteropolymers also called galactofucans/fucogalactans [34] and can be found in *Sargassum siliquosum*, with galactose and fucose accounting for 24.8% and 47.1% of monosaccharides, respectively [41]. Some fucoidans from *Undaria, Laminaria, Lobophora*, and *Sargassum* may contain fucose and galactose in comparable amounts [13,42,43,44,45]. Sulfated galactofucans structures have been associated with more promising bioactivities than fucans, with those from *Undaria pinnatifida* being the most studied [34].

**Figure 1:**
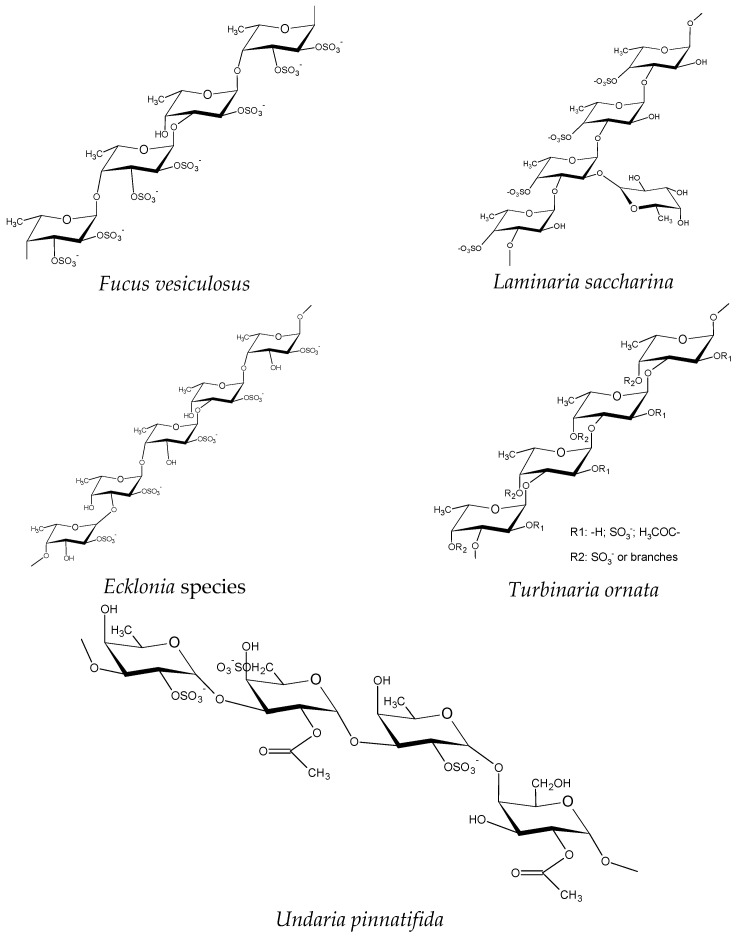
Examples of fucoidan structures.

Other unusual structures, i.e., with large proportions of mannose and uronic acids have been identified [32]. Fucoidans containing uronic acids have also been found [10] or with a high uronic acid content and mannose as a dominant sugar, followed by fucose, glucose, galactose, and some minor sugars of xylose and rhamnose [27]. Some representative examples are shown in Figure 1.

## 3. Chronic Inflammation

Under physiological conditions, the body activates the immune system to defend against an offending agent, such as bacteria, viruses, toxic chemicals, or an injury. First, the immune system responds by mobilizing inflammatory cells and releasing cytokines, which further boosts the presence of these inflammatory cells. As a result, a sequence of events is triggered involving inducers, sensors, mediators, and effectors [46]. The controlled inflammatory response is generally beneficial and aims to heal the attacked or injured tissue. However, it can become detrimental if not regulated, generating an acute inflammatory reaction or chronic inflammation [47].

Chronic inflammation could result from untreated or recurrent acute inflammation, exposure to toxins or irritant/foreign substances, autoimmune disorders, failure to adequately control pro-inflammatory inducers and mediators, some lifestyle factors (i.e., smoke, obesity, excessive or lack of exercise, and alcoholism), or age. Although chronic inflammation is less potent and harmful than acute inflammation in the short term, it causes accumulative damage in the long term, being the most significant cause of death in the world [48]. Therefore, it is considered a major contributor to several diseases. They include respiratory and neurodegenerative disorders like asthma and Alzheimer’s disease, respectively, cancer, type 2 diabetes and its related complications, cardiovascular diseases, and rheumatic disorders.

The inflammation present in a chronic status is characterized by a replacement of short-lived neutrophils observed in acute inflammation with an increase in the number of macrophages and lymphocytes [49,50], which release pro-inflammatory cytokines like IL-1, IL-6, and TNF-α and catabolic mediators (growth factors and enzymes such as metalloproteinases) that contribute to the progression of tissue and organ damage and aberrant repairing processes including fibrosis [51,52]. The release of reactive oxygen species is also observed in chronic inflammation-associated pathologies, participating in their development and progression [53,54]. Apart from leukocytes, circulating platelets can also contribute to inflammation by platelet aggregation, thrombus formation, and degranulation which releases inflammatory mediators and chemokines that stimulated infiltration of leukocytes, perpetuating the inflammation [55,56,57,58,59].

**Figure 2:**
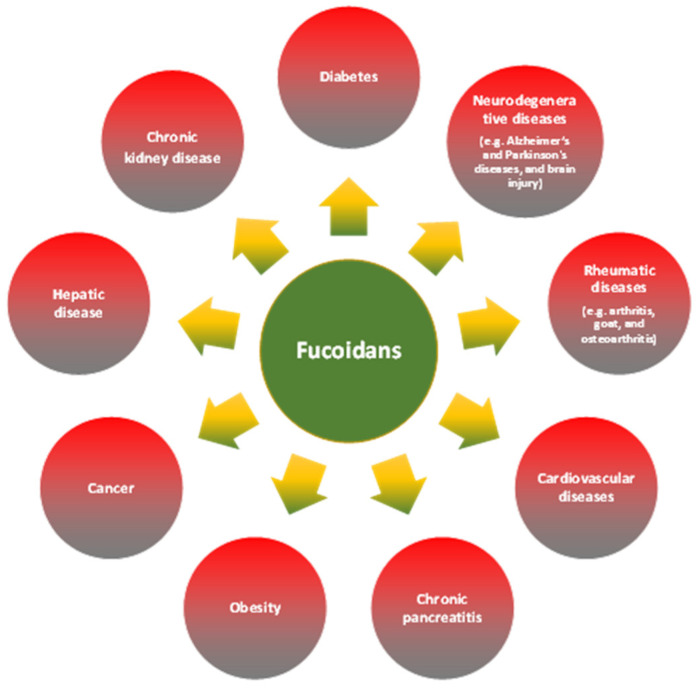
Disease conditions associated with chronic inflammation where protective effects of fucoidans have been observed [63,64,65,66,67].

In relation to treatment, there are different available pharmacological interventions presenting anti-inflammatory effects. The drugs are usually limited to specific diseases or group of diseases, as metformin for type II diabetic patients with dyslipidemia, or statins in people suffering cardiovascular pathologies. In contrast, other therapeutical strategies are based on the consumption of non-steroidal anti-inflammatory drugs (NSAIDs) or corticosteroids which are used in a wide range of conditions. Nonetheless, long-term therapy with any of these drugs could have side effects which could worsen the health of the patient [60,61,62]. In this regard, the therapeutic use of natural biomolecules, like fucoidans, have gained a great interest as an alternative and complementary therapy for the treatment of different diseases characterized by chronic inflammation, on the basis of its anti-inflammatory, antioxidant, and antitumor properties among others (Figure 2) [34,63].

## 4. Mechanisms of Fucoidan Actions

Fucoidans act on different stages of the inflammatory process, including blocking lymphocyte adhesion and invasion, inhibiting multiple enzymes, modulating inflammation-related gene expression, transcription factors, and adhesion molecules, inhibiting matrix metalloproteinases and complement cascade properties, and inducing apoptosis. Besides, they have also demonstrated to protect against inflammatory pathologies *in vivo* [7,22,35,68] as well as affect multiple therapeutic targets during the onset and development of inflammation-related diseases [20]. This section of the review aims to summarize the main signaling pathways mediating the anti-inflammatory effects of fucoidans.

### 4.1. NF-κB Signaling

NF-κB represents a family of inducible transcription factors that regulates a large array of genes involved in different processes of the immune and inflammatory responses [69]. NF-κB activation is induced by stress, cytokines, MAPKs, and nuclear hormone receptors among others. Under unstressed conditions, NF-κB stays in its inactive form in the cytoplasm bound to the inhibitor κB (I-κB) in a homo- or heterodimeric form, with p50 and p65 as the most common subunits. When a stress stimulus induces I-κB phosphorylation, the cytoplasmic subunits of NF-κB are released and translocate into nucleus where they exert their transcriptional activity. Hence, inhibition of NF-κB signaling is a putative mechanism underlying the downregulation of chronic inflammation. Likewise, different studies have shown that fucoidans modulate inflammatory responses thought inhibition of NF-κB [33,67,70]. For instance, fucoidans isolated using Viscozyme-assisted enzymatic extraction of *Sargassum coreanum* and showing high sulfate and fucose contents, and fucoidans from fermented *Sargassum fusiforme* obtained by ethanol precipitation, suppressed pro-inflammatory cytokine production (i.e., TNF-α, IL-1β, and IL-6) and NO by modulating NF-κB signaling in LPS-induced RAW 264.7 macrophages cells [71,72]. Similarly, a recent study demonstrated that fucoidans from *Undaria pinnatifida, Fucus vesiculosus*, and *Macrocystis pyrifera* can inhibit inflammatory responses and protect against mitochondrial dysfunction in cultured osteoarthritis chondrocytes by attenuating NF-κB nuclear translocation [73]. Interestingly, it has also been suggested that fucoidans are potential nutraceutical products against obesity-associated diseases and disorders via control of different signaling pathways, including NF-κB [74]. In addition, modulation of this pathway by extracted fucoidans has been associated with beneficial effects on pathologies such as renal fibrosis, diabetic nephropathy, and liver cancer among others [75,76,77].

### 4.2. MAPK Signaling Pathways

Mitogen-activated protein kinases (MAPKs) are a group of protein kinases that phosphorylate their own dual serine and threonine residues, or those found on their substrates, to regulate the activity of their target [78]. MAPKs include three protein groups—p38 kinases, c-Jun N-terminal kinases (JNK), and extracellular regulated protein kinase 1/2 (ERK1/2)—that are activated by different stimuli including pro-inflammatory mediators or oxidative stress [78,79]. These molecules play an important role in several cellular functions, but also up-regulate inflammatory responses inducing NF-κB activation and secretion of pro-inflammatory cytokines [78,79]. Several studies have reported that fucoidans isolated from brown seaweeds have the potential to inhibit MAPK phosphorylation [65,80,81]. Fucoidans obtained from *Saccharina japonica* demonstrated anti-inflammatory effects in vivo and in vitro in macrophages through the downregulation of MAPK expression (such as p38, ENK, and JNK) and NF-κB (such as p65 and IKKα/IKKβ) signaling pathways [33]. Low-molecular-weight fucoidans have been described to have a good anti-atherosclerosis effect by inhibiting p38 phosphorylation. Nonetheless, low molecular weight fraction (LMWF) fucoidans from *Undaria pinnatifida* have a strong immunological boosting effect through activation of TLR4 and its downstream MAPK and NF-κB signaling pathways [70]. An explanation of these opposing findings is given by Do et al., who observed a selective and cell-type-specific effect of fucoidans on the modulation of inflammatory responses in the brain and peritoneal macrophages, inhibiting or activating it, respectively, likely due to its dual effect on p38 activation [82].

### 4.3. TLR Signaling Pathways

Toll-like receptors (TLRs) are an important family of receptors that constitute the first line of defense system against microbes. Thus, they play crucial roles in the innate immune system by recognizing pathogen-associated molecular patterns [83]. Activation of TLRs in response to metabolites from diverse microorganisms triggers intracellular signaling cascades, including the pro-inflammatory transcription factor NF-κB [73,83]. Likewise, different findings have indicated that fucoidans could induce in vivo defenses against pathogenic microorganisms through this pathway [65,84,85]. A recent study suggests that fucoidans extracted from *Ascophyllum nodosum* prevent LPS-induced inflammation in macrophages by inhibition of TLR/NF-κB [86]. Interestingly, the extracts with lower molecular weights showed strongest effects in this study, indicating an influence of molecular weight on the anti-inflammatory activity of fucoidans [86]. Nonetheless, Nagahawatta et al. observed that fucoidans from *Ecklonia maxima* with similar molecular weights to those reported in the later study but higher sulfate contents than other extracts showed the best anti-inflammatory effect by attenuating TLR-mediated NF-κB/MAPK signaling [81].

### 4.4. TGF-β1 Signaling Pathway

Transforming growth factor (TGF)-β1 is an important pleiotropic cytokine with potent immunoregulatory properties that is produced by multiple lineages of leukocytes, stromal cells, and epithelial cells [87,88]. TGF-β first binds to the TGF-βR, which then primarily activates Smad transcription factors by phosphorylation and then the Smad complex translocates into the nucleus, and in turn regulates the transcription of target genes [88]. Nonetheless, Smad-independent pathways could also mediate TGF-β actions. TGF-β is involved in many pivotal physiological cellular processes, and has also been associated with inflammation, fibrosis, and pathologies such as cancer [87,88,89,90]. Fucoidans have been described to modulate TGF-β1 signaling pathways [66,90,91,92]. For instance, different molecular weight fucoidans from *Saccharina japonica* inhibited TGF-β1 induced epithelial–mesenchymal transition in mouse renal tubular epithelial cells [93]. Similarly, commercial fucoidans from *Fucus vesiculosus*, *Macrocystis pyrifera*, and *Undaria pinnatifida* showed different capacities to modulate pro-fibrotic processes in TGF-β1-activated synovial fibroblasts [90]. Fucoidan-functionalized micelles from *Fucus vesiculosus* exhibited excellent anti-tumor and anti-metastasis efficacy, inhibiting the expression of TGF-β [94].

### 4.5. JAK–STAT Signaling Pathways

The Janus kinase (JAK)–signal transducer and activator of transcription (STAT) pathway plays critical roles in orchestrating the immune responses, inducing transcription of inflammation-related genes, and polarizing T cells among other processes [95]. The binding of extracellular ligands to JAK receptors leads to intracellular receptor-associated JAK phosphorylation. Trans-phosphorylated JAKs then phosphorylate downstream substrates, including STATs. Activated STATs enter into the nucleus to regulate transcription of pro-inflammatory genes [95,96]. Interestingly, a growing number of findings indicate that fucoidans modulate inflammatory responses through this pathway [82,97,98]. For instance, sulfate-rich fucoidans isolated from *Saccharina japonica* inhibited LPS-induced production of various inflammatory mediators and pro-inflammation cytokines in macrophages through blocking the NF-κB, MAPK and JAK-2/STAT-1/3 signaling pathways [99]. Conversely, Yang et al. recently observed that the JAK–STAT pathway is critical for fucoidans to enhance antitumor immunity [100].

### 4.6. Nrf-2/Keap1 Signaling Pathway

Nuclear factor erythroid 2-related factor-2 (Nrf-2), a master transcription factor involved in antioxidant signaling and cell survival responses, regulates a wide battery of cytoprotective responses and protects against pathologies associated with oxidative stress and chronic inflammation such as metabolic, neurodegenerative, and other age-related diseases [59,101,102]. Under physiological conditions, Nrf-2 is generally located in the cytoplasm and binds to its inhibitor, Kelch-like ECH-associated protein 1 (Keap1), leading to its degradation. However, in response to oxidative or electrophilic stress, Nrf-2 dissociates from Keap1 and translocates to the nucleus to bind antioxidant-responsive elements in the promoter regions of its downstream antioxidant genes, including heme oxygenase-1 (HO-1) [59,101,102]. Different studies showed that fucoidans diminished oxidative stress and the expression of pro-inflammatory mediators by regulating Nrf2/Keap1 signaling [65,66,92]. A protective role of fucoidans from *Laminaria japonica* has been described in a model of cognitive dysfunction associated with chronic kidney disease in which the fucoidans ameliorated inflammatory responses and oxidative stress via GSK3β–Nrf2–HO-1 signaling [103]. Commercial and crude fucoidans from *Undaria pinnatifida*, *Fucus vesiculosus*, and *Macrocystis pyrifera* showed anti-oxidant and anti-inflammatory properties in an in vitro model of osteoarthritis by upregulating Nrf-2/HO-1 expression [73,104]. Similarly, low-molecular-weight fucoidans from *Sargassum confusum* suppressed inflammatory responses in keratinocytes via activating the Nrf-2/HO-1 signaling pathway [80]. In addition, Wang et al. recently observed that fucoidans isolated from fermented *Sargassum fusiforme* presented potent anti-apoptotic and antioxidant effects through upregulation of Nrf-2 levels. In an in vivo study of long-term alcohol-induced liver injury, fucoidan supplementation protected the liver from oxidative damage and hepatocytes from ferroptosis through upregulating the p62/Nrf2/SLC7A11 pathway [105].

### 4.7. Sirtuins

Sirtuins are a family of class III histone deacetylases, mediating the deacetylation of histones and non-histone proteins in an NAD+-dependent manner [106]. Sirtuins play a critical role during cell responses to a variety of stresses, such as oxidative stress, and are crucial for cell metabolism [106,107,108]. In humans, we can distinguish seven sirtuins (Sirt1–7). Sirt1, the best studied in the family, is a post-translational regulator that plays a known role in modulating inflammation [106]. However, a number of findings indicate that all SIRTs are involved in oxidative stress and its associated pathologies such as metabolic, cardiovascular, and neurodegenerative diseases [108]. Likewise, Akter et al. indicated that fucoidans elicits several biological responses, such as anti-inflammatory, antidiabetic, and anticancer responses by activation of Sirt6 [109]. Sirt1 has also been associated with the anti-diabetic and antifibrotic effects of low-molecular-weight fucoidans from *Sargassum hemiphyllum* and *Laminaria japonica* [92,110]. In this regard, the evidence suggests that Sirt3 is a key player for achieving the neuroprotective role of fucoidans through protection of mitochondrial function and modulation of gene expression, whereas Sirt1 appears to be associated with the regulation of glucose and lipid metabolism in the liver by these polysaccharides [111]. 

**Figure 3:**
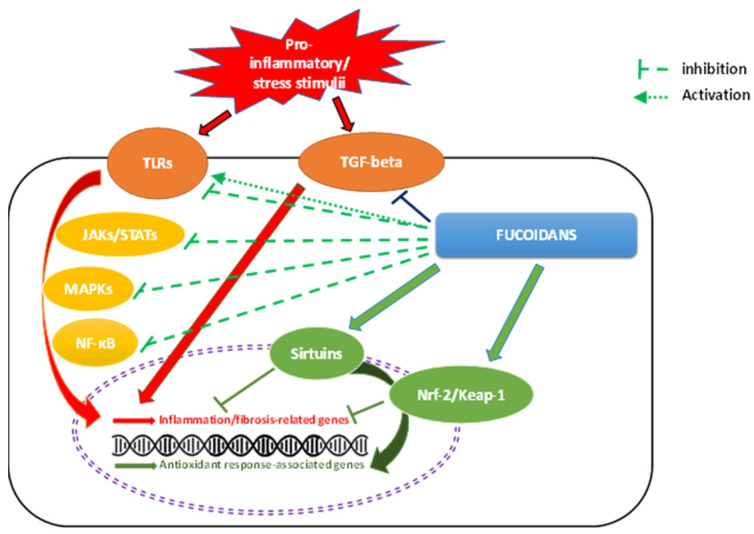
Anti-inflammatory effect of fucoidans. ERK, Extracellular signal-regulated protein kinase; IL, Interleukin; JAK, Janus kinase; JNK, c-Jun N-terminal kinase; Keap-1, Kelch-like ECH-associated protein 1; MAPK, Mitogen-activated protein kinase; NO, Nitric oxide; NF-κB, Nuclear factor kappa-B; Nrf-2, Nuclear factor-erythroid 2-related factor-2; ROS, Reactive oxygen species; STAT, Signal transducer and activator of transcription; TGF-β1, Transforming growth factor-β1; TLR, Toll-like receptor; and TNF-α, Tumor necrosis factor-α. Red lines with arrows show pro-inflammatory cascades involving activation of different pathways. Green lines with arrows represent anti-inflammatory actions of fucoidans.

In summary, pro-inflammatory and stress stimuli trigger inflammation and tissue damage by inducing production of ROS and the expression and release of pro-catabolic mediators (IL-1β, IL-6, TNF-α, NO,...) through activation of different signaling pathways such as TLR, NF-κB, MAPKs (JNK, p38, ERK), and JAKs/STATs. Fucoidans may attenuate the inflammation by blocking activation of these pathways and promoting anti-inflammatory and antioxidants responses mediated by sirtuins and Nrf-2/Keap1 pathways among others. Nonetheless, different findings indicate that fucoidan could also elicit inflammatory responses to enhance the antitumoral immunity or the response to pathogens (Figure 3).

## 5. Major Fucoidan Features Influencing Anti-Inflammatory Activity

### 5.1. Effect of SCP Sulfate Content

The sulfate content is one of the factors affecting fucoidan activity. Nagahawatta et al. reported that a purified fucoidan fraction from *Ecklonia maxima*, selected for its high sulfate content, suppressed the production of NO, prostaglandin E2, and pro-inflammatory cytokines (TNF-α, IL-6, and IL-1β), by inhibiting the nuclear factor kappa B (NF-κB) and mitogen-activated protein kinase (MAPK) signaling pathways in particulate matter-stimulated RAW 264.7 cells [81]. An optimal sulfate content has been suggested [112]. Wu et al. prepared polysaccharides with various sulfate contents by the sulfation and desulfation treatments of an original 386 kDa fucoidan with 31% sulfate [27]. The sample with 9% sulfate had the highest NO inhibition effects in LPS-induced RAW264.7 cells, while the sample with 51% sulfate had the lowest activity. Chen et al. found that TNF-α production was inhibited more efficiently by *Sargassum siliquosum* fucoidan fractions with higher sulfate contents [113].

### 5.2. Molecular Weight

Fucoidans present a wide variation in molecular weight, ranging from 21 to 1600 kDa, due to species variations and differences in the extraction and purification methods [114]. Low-molecular-weight fucoidans exhibit enhanced solubility and bioavailability and their interest for the development of pharmaceutical applications is increasing, although their long-term effects in the cell environment needs further evaluation [22]. However, there is no clear consensus on establishing the limits for high-, medium-, and low-molecular-weight fucoidans established as 100, 3.5, and 1 kDa, respectively [115], or as under 30 kDa [22] or 107.3-3.2 kDa [113].

Ahmad et al. confirmed that the 5–30 kDa subfraction from *Macrocystis pyrifera* reduced pro-inflammatory cytokine production (TNF-α, IL-1β, and IL-6) by human peripheral blood mononuclear cells and human macrophages (THP-1) more efficiently than fractions up to 600 kDa from this seaweed and from *Undaria pinnatifida*, *Fucus vesiculosus*, *Ascophyllum nodosum*, and *Laminaria japonica* [22]. All of them showed a carbohydrate content of 51–67% and sulfate content from 14 to 31%. Park et al. reported that in macrophage cell lines, the high-molecular-weight fraction (HMWF) induced the expression of inflammatory mediators, and enhanced the cellular migration of macrophages, whereas the LMWF did not. A daily oral dose of HMWF worsened the severity of collagen-induced arthritis and inflammatory responses in the cartilage and enhanced the production of IFN-γ; the LMWF had the opposite effect and reduced arthritis through the suppression of Th1-mediated immune reactions [115].

The depolymerization method is highly relevant. Park et al. suggested that the mild acidic method used to prepare low-molecular-weight fucoidans may change the chemical composition of the fucoidans [115]. Wu et al. found that acid digestion of fucoidans with molecular weights in the range of 386–1193 kDa yielded fractions with 2–106 kDa, which showed lower NO inhibitory activity than the original fucoidans [27]. Lahrsen et al. found stronger inhibitory activity for the smallest fucoidan fractions, 10.3 kDa from hydrothermal and 4.9 kDa from H_2_O_2_ degradation of a commercial *Fucus vesiculosus* fucoidan, even when both fucoidans and degraded fractions contained the same proportion of the constituents [40]. An optimal H_2_O_2_ concentration should be established, since the fractions obtained after depolymerization with 10–20% hydrogen peroxide were less active than those produced hydrothermally at 120 °C, whereas those obtained with 0.5–3.0% H_2_O_2_ yielded smaller and more active elastase inhibitors than those from hydrothermal treatments. These authors suggested that the FXII activating properties and the marginal anticoagulant activity of the fucoidan fractions do not have any clinical relevance. Additionally, Chen et al. reported the marked effect of hydrogen peroxide concentration on the molecular mass and the optimal inhibition of LPS-induced TNF-α production by the 3 kDa fraction from *Sargassum siliquosum* [113]. This fraction also contained a higher sulfate content.

The molecular weight could be also relevant for fucoidan absorption from different pharmaceutical preparations since low-molecular-weight (LMW) fucoidans may accelerate drug absorption and possess better access to cell surface receptors. Different studies evaluated the topical application of fucoidans (e.g., 49.8 kDa with immunomodulatory and anti-inflammatory actions on atopic dermatitis [116] and a 750 kDa *Fucus vesiculosus* fucoidan) which exhibited good skin-penetrating properties after topical application of an anti-inflammatory cream with efficacies comparable to diclofenac gel.

### 5.3. Other Factors

Wu et al. speculated that other factors besides molecular weight and sulfate content may also affect the anti-inflammatory activity of fucoidans, and suggested that the appropriate molecular weight and the existence of the triple helix conformation improved anti-inflammatory activity [20]. However, Cumashi et al. found that neither the content of fucose, nor the sulfate or the different structural features of fucoidans from different seaweeds affected their inhibition of leucocyte recruitment in an inflammation model in rats [117].

## 6. Extraction and Purification

The chemical composition of fucoidans, regarding monosaccharide type and content, and sulfate content and position, as well as the molecular weight and conformation are species-dependent [6,44,118] and are also determined by both biotic and abiotic factors (species, growth stage, part of the alga, seasonal and geographical conditions) and by the extraction and purification techniques [6,119,120].

The presence of other polysaccharides and hydrocolloids in brown seaweeds, alginates, and laminarans could limit the accessibility to solutes. Therefore, fucoidans are generally extracted in multistep processes using dilute acid or water at high temperatures for a long time. Under these conditions, excessive degradation and desulfation could occur. However, partial degradation of fucoidans can be desirable to obtain enhanced bioavailability and, in some cases, bioactivity. For this purpose, further depolymerization stages have been reported. Oligosaccharide fragments of fucoidan molecules were obtained by autohydrolysis [121] by physical, chemical or biological methods, such as radiation, acid hydrolysis, or enzyme hydrolysis [122].

Before extraction, seaweeds are conditioned to facilitate the destruction and degradation of the cell wall, increasing the surface area of the biomass with the extracting agent [123,124]. Alternatively, more complex stages, such as compression-puffing (140–220 °C for 10 s), or more simple methods such as direct extraction of algal pieces without prior conditioning pre-treatments, either fresh or frozen, or only cutting have been used.

In order to prevent the coextraction of low-molecular-weight compounds, colored substances, lipids, and lipophilic pigments, preliminary extraction stages can be performed. This pre-extraction stage has been frequently addressed with 70–96% ethanolic solutions at 25–80 °C [112,113,125,126], with acetone [10,127,128], or with solvent mixtures [118,123,129,130,131,132,133]. Alternatively, deoiling by sc-CO_2_ extraction was proposed [134,135]. Optionally, this stage was followed by formaldehyde/ethanol treatments to remove attached polyphenols [136,137]. Such stages are usually applied to dried seaweeds, but the extraction of frozen seaweed with a mixture of methylene chloride/ethanol has also been reported [138]. These pretreatments have been reviewed and compiled [123,139]. The seaweed to solvent ratio has to be defined to maximize the yield and concentration of the products, and solvent to seaweed ratios of 10–35 *w*/*w* or *v*/*w* (d.b.) are frequently reported.

### 6.1. Conventional Extraction

The extraction method should be selected to increase the extraction yield and preventing the possible structural alteration of the sulfated polysaccharides. The adequate operation conditions, including liquid to solid ratio, temperature, pH, extraction time, and number of stages, greatly influences the yield and composition of fucoidans [123], and determines the purification process. Examples of conventional techniques used for fucoidan extraction have been previously complied [120,139] and some examples are summarized in Table 1.

#### 6.1.1. Water Extraction

Water is an ideal solvent for fucoidan extraction in an organic-solvent-free extraction process approach [14]. A conventional method could rely on the use of hot extraction with tap or with distilled water at 70–90 °C during 2–6 h in 1–3 stages. Optimal temperatures and times should be selected to increase efficiency without negatively affecting the process. Operation at room temperature is possible, but longer extraction periods are required [140]. In order to enhance the yields and purity, dilute alkaline or acidic solutions have been proposed, but then the extracts need to be further neutralized to prevent polysaccharide degradation. In addition, intensification strategies such as ultrasound, microwave, or subcritical water extraction have been proposed.

#### 6.1.2. Alkaline Extraction

The use of CaCl_2_ solutions as solvent allows the selective precipitation of alginates during extraction [6,118,130,139,141]. These hydrocolloids are formed by mannuronic and guluronic acids and can gelate in the presence of Ca^2+^ or Mn^2+^. Extraction with aqueous calcium chloride solution yields polysaccharides with lower laminaran, uronic acid, and polyphenol content than those obtained by hot extraction [124]. When a simultaneous extraction of fucoidans and alginates occurs, the selective precipitation with CaCl_2_ allows the removal of alginate as a calcium salt, which could be separated by filtration [12,118,130,135,141] and the further addition of ethanol is useful for crude fucoidan precipitation [11,19]. Direct extraction with an alkaline treatment at room temperature has also been reported [142].

**Table 1:** Some examples of conventional extraction of fucoidan from brown seaweeds.

Aqueous Media	Seaweeds	References
Water, room temp., 2–24 h, 1–3 stages	*Fucus vesiculosus,* *Sargassum stenophyllum*	[44,140]
Water, 40–65 °C, 15 min,1 h, 1–2 stages	*Sargassum cristaefolium, Sargassum wightii, Undaria pinnatifida*	[12,126]
Water, 70–80 °C, 3–24 h, 1–3 stages	*Ecklonia cava,* *Fucus vesiculosus,* *Hizikia fusiforme,* *Saccharina japonica*	[18]
Water, 90–95 °C, 3–4 h	*Chnoospora minima,* *Halimeda discoidea, Halimeda gracilis*	[25]
Acidic Media		
0.05 M HCl, 25 °C, 2 h	*Saccharina japonica*	[135]
HCl (pH 2), 60–70 °C, 1–7 h, 1–9 stages	*Dictyota dichotoma, Laminaria cichorioides, Padina* sp., *Sargassum binderi*	[121,124,133,143]
30% HCl, 100 °C, 15 min	*Cladosiphon okamuranus*	[144]
0.01 M HCl or 0.001–0.1 M H_2_SO_4_, 60–80 °C	*Fucus vesiculosus*	[145]
0.03–8 M HCl (pH 2), 90–100 °C, 0.25–4 h	*Sargassum* sp., *Sargassum fulvellum*	[131,146]
0.15 M HCl, 45 °C, 3 h, 4 stages	*Sargassum* sp.	[141]
McIlvaine’s buffer solution (pH 4.0), 60 °C, 3 h	*Sargassum* sp.	[11]
HCl (pH 2), room temp., 24 h	*Undaria pinnatifida*	[147]
Alkaline Media		
1–2% CaCl_2_, room temp., 45–85 °C, 5–24 h, 1–6 stages	*Ascophyllum nodosum*, *Fucus evanescens*, *Fucus dischitus*, *Fucus serratus*, *Fucus spiralis*, *Fucus vesiculosus*, *Laminaria digitata*, *Sargassum binderi*, *Sargassum* sp., *Undaria pinnatifida*,	[117,118,124,129,132,133,141]
4 M KOH, 10 mg NaBH_4_, room temp.	*Sargassum stenophyllum*	[142]

#### 6.1.3. Acid Extraction

Seaweed polysaccharides can be efficiently extracted with dilute acids at room or higher temperatures. However, depolymerization could occur under severe conditions. Therefore, adequate selection of the operational conditions (extraction pH, time, and temperature) is required to tune the molecular weight, monosaccharide composition, and sulfate content, which determine the bioactivity of the products [131,141].

The acid concentration can affect the extraction efficiency. Most studies report the use of mild acidic solutions (0.01–0.1 M) of HCl [124,131,148,149]. Alternatively, 1% H_2_SO_4_ was selected for *Undaria pinnatifida* [41]. Temperature is an important variable usually fixed in the range of 60–90 °C. Lorbeer et al. [148] proposed a milder process at 42 °C, pH 1.0, and 159 min for sequential extraction of fucoidans and alginates of *Ecklonia radiata* in an industrially relevant context. The increase in temperature from 35 to 70 °C during HCl extraction caused a significant molecular weight reduction [114]. The hydrolysis into lower-molecular-weight fucoidan occurring under higher temperatures and in mild acidic media could be of interest to enhance some biological properties [145,150], but excessive depolymerization and desulfation is not desirable [27]. Classical procedures usually require prolonged times using diluted acids, water, or 2% aqueous calcium chloride. Conventional water extraction yields 1.2–9.8% [12,14,44], between 2–22% for acid extraction [14,41,131,143], and 4.9% for alkaline extraction [142]. Mild acid hydrolysis can also be applied to the crude fucoidans or to fractions for depolymerization purposes compared with other available techniques using chemicals, e.g., radical, radical and acid, fucoidan-degrading enzymes [151].

### 6.2. Alternative Extraction Procedures

The increasing consciousness regarding safety, health, and environment is leading to the development of more efficient and eco-friendly extraction and purification processes [123,139]. Innovative emerging and scalable technologies are also being incorporated for the extraction of seaweed polysaccharides, with advantages derived from the reduced amounts of solvent, time, and energy consumption, as well as lower emissions, and the increased safety and product quality [123]. The major advantages are summarized in Table 2. Different techniques, including pressurized solvents, and assistance by enzymes, ultrasound, and/or microwaves proved successful to obtain fractions with conserved sulfation degrees [123].

#### 6.2.1. Ultrasound-Assisted Extraction

Ultrasound (US) waves with frequencies in the range 20 to 100 MHz are used in chemistry. The propagation of ultrasound waves in an elastic medium induces a series of compression and expansion cycles, leading to production, growth, and collapse of bubbles in a phenomenon known as cavitation. The application of US offers different beneficial effects on solid–liquid extractions, derived from combined physical and chemical mechanisms. Different physical effects can favor the extraction process, including the erosion induced by shear forces caused by cavitation at the vicinity of solids, the destruction of cell walls, increased solvent penetration into the solid matrix, and the macroturbulences and micromixing in the liquid medium. Different chemical effects are observed, mainly derived from the high pressures and temperatures in the bubbles, generating free radicals [152].

Both baths and probes can be scaled up and allow batch, semicontinuous, and continuous operations. The action of ultrasound is affected by the medium (particle size, liquid:solid ratio, temperature, presence of gases) and the equipment (power, frequency, intensity, shape and size, and time of sonication) characteristics [153]. The power intensity per area (W/cm^2^) or per volume (W/cm^3^) can be an adequate criterion for comparative and scaling up purposes [154,155]. Temperature is highly influential since an increase can enhance the solubility, but should be chosen with care to control cavitation and to avoid degradation of thermolabile compounds and undesirable reactions [155]. Other relevant variables are the frequency as well as the solid/liquid ratio and particle size of the material.

Ultrasound offers reproducibility, mild operation conditions, reduced thermal gradients, and more effective mixing, resulting in enhanced extraction yield and rates and a reduction in extraction time, equipment size, energy, and use of solvent. The composition and molecular weight of fucoidans differ with extraction time and should be optimized to enhance the sulfate content. This technique can be combined with conventional and/or innovative extraction technologies [139,147].

Ultrasound assistance was used to extract phenolics and fucoidans from *Ascophylum nodosum* during acid extraction [156] and fucoidans from *Undaria pinnatifida*, which resulted in increased yield and decreased extraction time [147]. This stage could also be proposed as a pretreatment, as reported from *Sargassum polycystum* (amplitude 80%, 15 min) before acid extraction [157]. Following ultrasonication, the average molecular weight of the fucoidans decreased [158] and the anti-inflammatory action of *U. pinnatifida* [147] and the immunomodulatory potential of *Hizikia fusiforme* [5] fucoidans were enhanced. This technology has also been used to depolymerize already extracted polysaccharide fractions [122,139,147].

#### 6.2.2. Microwave-Assisted Extraction

Microwave radiation equipment for domestic and commercial use operate at 2450 MHz, whereas industrial microwaves operate at 915 MHz. This radiation, generated by an electric field and a magnetic field oscillating perpendicularly to each other, causes heating by ionic conduction and dipole rotation. Microwave heating induces a sudden increase in temperature of the intracellular liquids and water evaporation, dramatically increasing internal pressure, inducing cell wall degradation and the release of intracellular contents. In addition, the dipole rotation of the molecules induces disruption of the solute–matrix interactions [159].

The particle size and liquid to solid ratio should be optimized for each material, and power has to be selected to maximize yields and selectivity of the target solutes. The operation temperature influences the product distribution: fucose was the main monosaccharide of *A. nodosum* fucoidans extracted at 90 °C whereas glucuronic acid was the main monosaccharide extracted at 150 °C [160,161]. Under pressurized conditions, short times (30 min) can be operative [161]. For the extraction of fucoidans from *E. radiata*, classical extraction conditions (HCl pH 2, 60 °C in 6 min) were optimal since at prolonged times undesirable declines in fucose and sulphate content, increases in laminarin, and reductions in the molecular weight of the fucoidans occurred [148].

Microwave radiation can involve desulfation and favor crude fucoidan depolymerization. Navarro et al. [162] reported 60–93% sulfate removal after 1 min of microwave radiation and a moderate depolymerization, but the integrity of the polysaccharides was not affected. It has been suggested that during the heat treatment of *U. pinnatifida*, rather than polymeric degradation, a disruption of the secondary interactions between fucoidan polymers occurred, which facilitated a better dissolution. The MW markedly decreased from 23,600 to 2400 kDa after 30 s, whereas only a slight decrease to 1900 kDa took place up to 90 s and to 500 kDa after 120 s. The authors found that the molecular weight after 30 s of microwave heating was lower than with boiling water for 15 min (5200–5900 kDa) [150]. Application of microwave irradiation has been described to aid in the depolymerization of previously extracted fucoidans from *Sargassum muticum* [122].

Microwave-assisted extraction requires lower amounts of solvent and provides improved yields, which shortens time and lowers energy requirements allowing reduced equipment size in scalable processes [163,164,165]. Operation in combination with other techniques, such as high pressure, vacuum, and ultrasound [164,166] has been reported.

#### 6.2.3. Enzyme-Assisted Extraction

The hydrolytic action of enzymes causes degradation or disruption of cell walls and membranes, thus being a useful tool to aid in the extraction of seaweed components found intracellularly in cytoplasm and not accessible to solvents in a conventional extraction [167]. Major factors affecting this process include the particle size, solid to water ratio, enzyme activity and concentration, temperature, pH, and time. Enzyme activity or commercial complexes of hydrolases are more effective to achieve cell wall disruption. Most studies have reported on the use of commercial food-grade enzymes, mainly amylases [168] but also proteases [17,169], developed for the extraction of natural products from terrestrial plant material [170]. The lack of fucoidanase action makes them suitable for these uses [123]. Even brown seaweeds, which have low protein content, the use of proteases favored the concentration of sulfated sugars in the crude extract, attaining comparable values as those found in extracts after hot water and with ultrasound-assisted extraction [171]. Since proteins could affect the purity level of the obtained polysaccharides, the use of alcalase, after a previous cellulase digestion, was used to assist the extraction of proteins [136]. Additionally, crude enzymes can be beneficial, such as those from *S. oneidensis* PKA 1008 that were used to enhance the polysaccharide degradation of SP and its anti-inflammatory effects in RAW 264.7 cells [28]. Alternatively, the hydrolysis could be addressed with microbial transformations. Wang et al. [172] isolated a 213 kDa fucoidan from fermented *Sargassum fusiforme*. This product inhibited LPS-induced nitric oxide production and reduced the prostaglandin E2, interleukin-1 beta, tumor necrosis factor-alpha, and interleukin-6 levels in RAW 264.7 cells and reduced reactive oxygen species, cell death, and NO levels in LPS-treated zebrafish.

Lee et al. [18] obtained fractions from 18 to 359 kDa in size after enzymatic extraction of *Ecklonia cava.* Hwang et al. [173] obtained a LMW fucoidan of 0.8 kDa with anti-inflammatory effects when combined with fucoxanthin coated with polysaccharides in a lipopolysaccharide-induced inflammatory Caco-2 cell line co-cultured with *Bifidobacterium lactis*. These compounds activated probiotic growth and reduced the inflammation of intestinal epithelial cells by enhancing the barrier and immune function against the lipopolysaccharide effect, inhibiting IL-1β and TNF-α, and promoting IL-10 and IFN-g.

If the enzymatic degradation of sulfated polysaccharides preserving the sulfate groups is desirable, the application of fucoidanases, α-L-fucosidases, and galactosidases could be selected. The enzyme to substrate ratio requires optimization since this factor significantly affects the efficiency and costs. Values in the range of 0.2–3.0% have been reported [170,171,174,175]. Different variables, relevant in conventional extractions, are also determinants in enzyme-assisted processes. Mechanical pretreatments favor the accessibility of the enzyme to the substrate. The liquid-to-solid ratio during enzyme digestion needs proper optimization for both the hydrolytic reaction and the mobility of the enzymes and products. pH and temperature are enzyme-dependent, with optimum pH (3.8–8) and temperature (40–60 °C) for enzymes such as amyloglucosidase, agarase, proteases, carragenanase, cellulases, β-glucanases, and xylanase having been reported [171,174].

The milder temperature and pressure conditions, the non-toxic and food-grade characteristics of enzymes, and the possibility of using them in large-scale processes are advantageous aspects compared to conventional technologies. The development of efficient strategies to recycle them should be considered [123].

Enzymatic extraction of *Ecklonia cava* may be more advantageous than water extraction, enhancing the extraction yields, the fucoidan content, and also the fucose and sulfate contents [18]. Commercial proteases and carbohydrases significantly improved biomass yield by 2–3 times over that achieved with water, and the extracts showed a variety of biological activities [42,175,176,177]. Enzyme-assisted extraction is a green method with improved efficiency over water extraction and maintains the sulfate content [19,25]. In some cases, the incorporation of carbohydrases and proteases during the extraction process had little or no impact on total sugar yield from *Ecklonia radiata*, although the molecular weight profile was reduced by 20–50% compared to control extractions [170]. Despite the lower yields, *Sargassum* sp. fucoidans that had been extracted by papain showed a lower content of fucose than that extracted by 0.15 M HCl or an alkaline solvent (CaCl_2_). However, fucoidan obtained by acidic solvents could result in the simultaneous extraction of undesirable products such as alginic acid and metals [141].

In many cases, the use of enzymes has been proposed to lower the molecular weight during extraction and also as a depolymerization stage. Kim et al. [178] used a fucoidanase isolated from *Pseudoalteromonas* sp. to hydrolyze a commercial high-molecular-weight fucoidan and after ultrafiltration they selected an 8 kDa fraction with antiphotoaging properties on UVB-irradiated skin damage, which could be the result of the cooperative interactions of antioxidant, anti-inflammatory, and MMP-inhibiting effects.

#### 6.2.4. Subcritical Water Extraction

Pressurized hot water extraction or low polarity water extraction is based on using only water as the solvent and operating between 100 °C and 1 bar and the critical point, at 374 °C and 221 bar. Under these subcritical conditions, solvent viscosity, density, and dielectric constant are markedly reduced, but the ionic product is increased. These characteristics convert subcritical water in a solvent of compounds with lower polarity than those soluble at lower temperatures, requiring lower solvent volumes and shorter times with benefits derived from the oxygen- and light-free environment [15,179]. It has been proposed for both the extraction and depolymerization of polysaccharides [180]. Furthermore, the selective extraction of crude fucoidans is also favored, lowering the protein contamination in comparison to acid and alkaline extraction [135].

Pressure is important to maintain the solvent in a liquid state; a minimum liquid to solid ratio is needed, but temperature and time are the most influencing variables and, in semi- and continuous operation, flow rate should also be considered. Adequate selection of temperature allows modulation of water polarity to solubilize more apolar molecules to allow for hydrolysis and to form novel compounds [181]. Subcritical water extraction proved suitable for the solubilization and depolymerization of fucoidan fractions [180]. The optimal temperature depended on the operation mode (isothermal or non-isothermal) and on the seaweed species. Maximum fucoidan extraction yield during non-isothermal conventional heating was obtained during heating up to 170 °C for *Sargassum muticum* [180], up to 140 °C for *Undaria pinnatifida* [134,182], or during isothermal heating in autoclave at 120 °C for 3 h [131,183]. Increasing the subcritical water extraction temperature and time lowered the fucoidan yields, especially operating at 180 °C and 210 °C, due to degradation. However, higher severity could favor the extraction of phlorotannins, found at higher concentrations compared to extracts from conventional hot water extraction [184,185]. Morimoto et al. [186] confirmed that hydrothermal treatment at 140 °C allowed fucoidan depolymerization without causing desulfation, being more rapid than aqueous processing at room temperature for prolonged times (several days) [125], an alternative that can also cause desulfation [149]. Vaamonde-García et al. [104] reported the attenuation of osteoarthritis associated anti-inflammation by fucoidans obtained from the crude extract by pressurized hot water extraction from *Sargassum muticum* and *Undaria pinnatifida*. The further depolymerization of subcritical water extraction fucoidans has been reported using ultrasound-assisted extraction [122] or H_2_O_2_ hydrolysis from *Sargassum* [26].

When selecting the intensification strategy, the influence on the composition and structural features should be considered. A compilation of the different techniques and their influence on the fucoidans’ characteristics in relation to their anti-inflammatory properties is shown in Table 3. Combinations of strategies can provide synergistic effects, and mixed processes have been reported for hydrothermal treatments with microwave heating in closed systems [134], microwaves, and ultrasound to combine the effects of enhanced heating and mass transfer, and the ultrasonic assistance is well known to enhance the enzyme activity. Comparisons among different experimental studies are difficult, and further studies are needed, but as a general trend, the different conventional and emerging extraction and depolymerization techniques can be adequately modulated to obtain crude extracts and fractions with low-molecular-weight and medium-high sulfate content, which can exert anti-inflammatory actions on different in vitro and in vivo models.

## 7. Coatings and Micro- and Nanoencapsulation

Fucoidans exhibit anti-inflammatory activity in both oral and topical applications [193], but its targeted delivery may be limited by its larger molecular size [66]. It has been suggested that encapsulation in nanoparticles could enhance some properties due to the increased permeability [48]. In addition, fucoidans can be incorporated in functionalized biomaterial scaffolds with good biocompatibility, biodegradability, and mechanical strength, useful for drug release, disease treatment, and for tissue repair and regeneration. Some recent examples can be used to illustrate the potential of fucoidans in different formulations. Wardani et al. [197] have reported the antioxidative and anti-inflammatory effect of fucoidan nanoparticles against nephropathy of streptozotocin-induced diabetes in rats. At a dose of 300 mg/kg BW, there was decreased BUN, creatinine, MDA, IL-6, and TNF-α levels, but increased SOD and GPx expression as compared with the streptozotocin group. Shin et al. [198] developed fucoidan-coated polymeric nanoparticles as renal IR-targeting nanotherapeutics that exerted anti-inflammatory and antiapoptotic effects by suppressing the generation of ROS and the expression of proinflammatory cytokines.

Naturally occurring biopolymers can be used as scaffolds for cartilage tissue engineering due to their anti-inflammatory, biocompatibility, biodegradability, low toxicity, and plasticity properties. Sumayya and Muraleedhara [199] have designed cross-linked bio-composite scaffolds composed of hydroxyapatite, alginate, chitosan, and fucoidan. These biocomposites inhibited the production of ROS, suppressed NF-kB translocation to the nucleus, and inhibited the production of inflammatory mediators.

Liu et al. [200] prepared stable and uniform fucoidan nanomicelles loaded with cannabidiol to treat oral mucositis based on its high binding affinity for P-selectin. Their local or systemic administration in vivo enhanced the retention and anti-inflammatory effect of cannabidiol, accelerating healing and inhibiting Ly6G infiltration and NF-κB nuclear transcription.

Hao et al. [201] prepared fucoidan-based hydrogels with tunable microporous architecture, swelling, and biodegradable properties via a facile chemical cross-linking approach in an alkali/urea aqueous system. The hydrogels were cytologically, histologically, and blood-compatible and, after subcutaneous implantation in rats, inhibited the inflammatory response of surrounding tissues.

Obluchinskaya et al. [138] proposed *Fucus vesiculosus* fucoidans for the formulation of non-irritating creams with good spreadability, washability, and colloidal stability. The formulation was stable and provided high release after storage for 1 year and topical application in rats dose-dependently inhibited carrageenan-induced edema with comparable efficacy to diclofenac gel.

Yu et al. [202] confirmed the potential of fucoidans in the treatment of intervertebral disc degeneration. Its incorporation into a biocompatible poly (ether carbonate urethane) urea nanofibrous scaffold reduced the inflammation and oxidative stress caused by lipopolysaccharide, lowering the gene expression of Il 6 and Ptgs2 and protein expression of genes related to the degradation of the extracellular matrix. *In vivo*, it promoted ECM deposition to maintain the height, water content, and mechanical properties of intervertebral discs.

Chen et al. [203] reported that topical application of *Cladosiphon okamuranus* fucoidans on a DNCB-induced mice model promoted skin repair, reduced immunocyte proliferation, and decreased serum IgE level, down-regulated AD-associated cytokines, and up-regulated TGF-β1 level. Regulation of systemic immunity was also observed as well as significant improvement in atopic dermatitis (AD) in both in vitro and in vivo models.

## 8. Conclusions and Future Trends

Sulfated polysaccharides from brown seaweed are attractive bioactives with a variety of biological properties that can be recovered from widely available, renewable sources. However, the variability in the sources and processing can influence the composition and structure of the fucoidans, particularly the degree of polymerization and the sulfation degree. Therefore, careful optimization of conditions is recommended, especially during the incorporation of innovative extraction methods, which allow a more efficient mass and energy transfer and can offer technical and economic advantages over classical extractions. In addition, the detailed chemical characterization of fucoidans and a better understanding of their bioavailability and multifunctional actions in different in vitro and in vivo systems is also required for the development of supplements, nutraceuticals, or drugs to delay or prevent chronic inflammation and its associated diseases.

A challenge in exploiting the benefits of fucoidans is the presence of pyrogenic agents (endotoxins), especially when parenteral administration is required. Ahmad et al. [22] recommended the use of depyrogenated extracts to elaborate the dose–response curves. Depyrogenated products showed better anti-inflammatory properties than the original fractions, since they interact with cell surface receptors more effectively at lower concentrations, but these results must be checked in the pre-clinical models. These advances in basic and applied knowledge will be determinant for the development of potential uses in medicinal, food, and cosmetic applications. 

## Figures and Tables

**Table 2:** Advantages and limitations of conventional and emerging techniques for the extraction of fucoidans.

Extraction Technology Advantages Disadvantages	Challenges	Effects on Fucoidan Features and Anti-Inflammatory Properties
Conventional solvent extraction		
Simple and established	Prolonged time High temperatures High energy consumption Chemicals Degradation of products	Excessive degradation under severe conditions Desulfation and depolymerization
Subcritical water extraction		
Short processing time High extraction yields Selectivity by modulating operational conditions	High capital cost Degradation of products Undesirable byproducts	Simultaneous extraction and depolymerization Maintains structure and sulfation Further processing with other technologies
Enzyme assisted extraction		
Mild conditions Low energy requirements Selective Enhanced extraction yields	Slow process Availability and cost of enzymes Enzyme recycling and reuse strategies	Depolymerization Maintains sulfation degree
Ultrasound assisted extraction		
Simplicity Mild temperatures Short time Decreased use of solvent Scalable Reduced equipment size Low energy, costs, and risks	Localized heating and non-uniform conditions Radical formation and possible degradation Noise pollution	Reduction in molecular weight
Microwave assisted extraction		
Rapid heating Short extraction times Increased yield	High energy needs Thermal degradation of some components	Degradation of structure sulfate

**Table 3:** Examples of extraction and purification methods to obtain fucoidans with anti-inflammatory properties.

Seaweed/Processing	Model System and Anti-Inflammatory Actions	Reference
*Chooospora minima* Pre-Ext: 95% E; 10% FMD in 95% E, 8 h, 37 °C, E wash Ext: Celluclast (0.5% E/S), pH 4.5, 50 °C, 24 h Alcalase (0.5% E/S), pH 8.0, 50 °C, 24 h Neutr., 5 M CaCl_2_ alginate pptn; conc., 95% E pptn Purif: AEC 63.5 % carboh.; 34.1% sulf.; 0.2% prot.; 0.4% polyph.	LPS-stimulated RAW cells Reduced activity of iNOS and NO levels. Downregulation of PGE2, reduction in COX-2 levels. Downregulation of pro-inflammatory cytokines TNF-α, IL1β, and IL6	[187]
*Ecklonia maxima * Pre-Ext: 95% E, 10% FMD in 95% E, 8 h, 37 °C, E washing Ext: Celluclast (0.5% E/S), pH 4.5, 50 °C, 24 h Alcalase (0.5% E/S), pH 8.0, 50 °C, 24 h Neutraliz., 5 M CaCl_2_ alginate pptn; conc.; 95% E pptn Purif: AEC 51.4% carbohyd.; 39.8 % sulf., 0.5% prot.; 0.3 % polyph. 81.8% Fuc, 0.33 % Rha, 0.04% Ara, 14.7%, Gal, 3.1% Man	Particulate matter-stimulated RAW 264.7 cells Suppressed production of NO, prostaglandin E2, and pro-inflammatory cytokines (TNF-α, IL-6, and IL-1β) by inhibiting the NF-κB and MAPK signaling pathways	[112]
*Ecklonia cava* Ext: Celluclast, 1%, 50 °C, 24 h E pptn; C; 4 M CaCl_2_ alginate pptn; cetylpyridinium chloride pptn, resolubilized with 3 M CaCl_2_; E pptn; Dl, FD 51.8% carbohyd.; 20.1 % sulf.; 11.3% uronic ac.; 8.9% protein 61.1% Fuc, 3.9% Rha; 27.2 Gal; 0.8 % Glu; 7.0% Xyl	Tail-cutting-induced and LPS-induced zebrafish Inhibition of ROS and NO generation	[188]
*Ecklonia cava * Ext: Glucoamylase, optimal pH and T, 1% (*v*/*w*), 24 h UF (50 kDa), E pptn Purif: AEC and GPC Fuc:Rha:Gal:Glu:Man:Xyl ratio 82.1:0.3:12.2:0.22.2:2.2 Sulf: Total sugars 0.92:1.38, 103 kDa	LPS-stimulated RAW 264.7 cells Inhibited NO and PGE2 production, and suppressed iNOS and COX-2 expression	[17]
*Fucus vesiculosus* Pre-Ext: MC:E Ext: US, 5% E, 40 °C, pH 3-4, 4 h; centr; Dl; FD 79.5% carbohyd., 27.0% sulf., 0.7% uronic ac. Fuc:Gluc:Gal:Xyl:Man:Ara (mol) 1.0:0.16:0.05:0.09:0.03:0.03; 735 kDa	Topical application in rats Inhibited carrageenan-induced edema with comparable efficacy to diclofenac gel	[138]
*Hizikia fusiforme * Ext: Celluclast (5% enzyme), pH 4.5, 50 °C, 24 h; E pptn Pur: AEC; Dl, FD 71.8% carbohyd., 27.2% sulf.; 102.67 kDa 79.2% Fuc, 18.1% Man, 2.1 % Rha, 0.4% Arab, 0.2% Glu	UVB-induced photodamage in vitro in human dermal fibroblasts and in vivo in zebrafish Improved collagen synthesis, inhibited intracellular collagenase, and reduced expression of matrix metalloproteinases and pro-inflammatory cytokines	[189]
*Kjellmaniella crassifolia * Ext: cellulase and pectinase; Filtr, E pptn, washed, FD	Gastric protective effects. Suppressed aspirin-induced NF-κB activation via stabilization of IκB-α	[190]
*Laminaria japonica* Ext: 30 mM H_2_O_2_ and ascorbate 1:1, 2 h Dl (3.6 kDa); E pptn 28.7% fuc; 3.6% uronic ac., 30.1% sulf., 6.5 kDa	Non-alcoholic fatty-liver disease in obese diabetic rats Reduced expression of TNF-α, MCP-1, and NF-κB. Down-regulation of pro-inflammatory cytokines and transcription factors, and up-regulation of adiponectin	[110]
*Lobophora variegata * Pre-Ext: A, 60 °C, 18 h Ext: Maxatase, A (1 vol) fractionation, molec. sieving, IEC; MeOH pptn, D, Fuc:Gal:Sulf (molar) 1:3:2	Ear swelling caused by croton oil Inhibited leukocyte migration to the inflammation site	[169]
*Padina commersonii* Frozen samples Ext: Celluclast Purif: DEAE-cellulose; Dl	LPS induction in RAW 264.7 macrophages Inhibited TLR2/4 and MyD88 transcriptional activities. Reduced the transcriptional activities of NF-κB signal transduction. Inhibited cytokine and NO secretion	[191]
*Saccharina japonica* Pre-Ext: 95% E, 2 h, 40 °C Ext: W (1:30, *w*/*v*), 120 °C, 2 h, C, 2% CaCl alginate pptn; C, conc.; Dl (3.5 kDa), FD Purif: AEC (DEAE) 56.5 % polysacch., 30.7% sulfate 79.5 % Fuc; 16.8 % Gal; 0.8 % Rha, 1.1% Xyl, 1.8% Man	LPS-induced RAW264.7 cells Decreased the production of NO, TNF-α, IL-1β and IL-6. Down-regulated expression of MAPK and NF-κB pathways. in vivo LPS-induced zebrafish Reduced cell death rate and production of NO and ROS	[192]
*Saccharina japonica * Pre-Ext: 95% E, 40 °C, 2 h Ext: W, 120 °C, 2 h C, 2% CaCl_2_ alginate pptn, conc., Dl, FD Purif: AEC, Fuc:Gal 79.2:20.8; 11.46% sulfate	RAW264.7 cells Reduced NO. Down-regulated MAPK (p38, ENK and JNK) and NF-κB (p65 and IKKα/IKKβ) pathways. Zebrafish Reduced cell death rate, inhibited NO, and decreased ROS	[33]
*Sargassum cristaefolium* Pre-Ext: (Compressional-puffing) 99% E, 25°C, 4 h Ext: SWE, 121°C, 20 min, Dl Pur: AEC, Dl, FD Depol: 1 M H_2_O_2_, 60 °C, 1 h, C, filtr., 3.2 kDa	LPS-stimulated RAW264.7 and HaCaT cells Inhibited COX2 and p38 UVB-induced damage in Hs68 cells and in rats After stimulation with LPS, TNF-α, and IFN-γ, improved redness and swelling caused by UVB irradiation	[26]
*Sargassum cristaefolium* Pre-Ext: E defatted with ethanol Ext: W, 100 °C, 1 h 95% E pptn; 75% E, 2 stages; FD	LPS-induced RAW264.7 cells Suppression of induced p38, ERK1/2, and JNK phosphorylation. Inhibited NO secretion. Down-regulated iNOS expression by inhibiting MAPK and NF-κB pathways	[27]
*Sargassum fusiforme * Pre-Ext: 80% E Ext: 0.01 M HCl 4 M CaCl_2_ alginate pptn., Dl, Conc, 95% E ppt, deproteinizat. Purif: AEC 64.1 % carbohyd., 28.2% uronic ac., 5.4% sulf. Man:Fuc:Rha:GlcA:Xyl:Gal:Glu (mol) 26.9: 21.5: 18.5: 9.9: 9.7: 7.7: 5.8	Binding of P-selectin to HL-60 cells Disrupted P-selectin-mediated cell adhesion and rolling and blocked the interaction between P-selectin and its physiological ligand PSGL-1	[20]
*Sargassum fusiforme* Pre-Ext: 90% E reflux Ext: W, 70 °C, 2 h, 2 stages, C, conc., UF, FD Depol: HCl (pH 2.0), neutr., E pptn; A and E washing; D 98.1% purity, 22% Fuc, 22.8% sulfate	LPS in normal human colonic epithelial cells in vivo experiments in mice Restored the diversity of gut microbial composition. Reduced incidence of tumors in mice. Decreased TNF-α, IL-6, and IL-1β expression levels	[31]
*Sargassum fusiforme* Ext: 0.4% citric ac., 60 °C, 120 min, homogeniz., neutr., steriliz., inoculated 5% *Lactobacillus rhamnosus,* 37 °C, 48 h, C, E pptn Purif: AEC 71.8% carbohydrate, 27.2% sulfate, 102.7 kDa 79.2% Fuc, 2.1% Rha, 0.2% Glu, 18.1 % Man, 0.4% Ara	LPS-stimulated RAW 264.7 cells Inhibited TNF-α, NO, PGE2, IL-1β, and IL-6 production. Improved viability. Suppressed the expression of COX-2 and iNOS by regulating the NF-κB pathway in vivo zebrafish Reduced ROS	[189]
*Sargassum horneri* Pre-Ext: 95% E; 10% FMD in E; 3 h; 95% E washing Celluclast, 50 °C, 8 h FD, Centr, E pptn Pur: AEC; Dl sulfated mannofucans 45 kDa	Fine-dust on skin inflammation in HaCaT keratinocytes Recovery of skin barrier dysfunction. Lowered ROS levels. Down-regulated TNF-α, IL-1β, -5, -6, -8, -13, interferon-γ, and chemokines. Inhibited mitogen-activated protein kinase and NF-κB pathways	[168]
*Sargassum hemiphyllum* Ext: W, 100 °C, 30 min C, FD	Lipopolysaccharide-activated RAW 264.7 cells Reduced secretion profiles of IL-1β, IL-6, TNF-α, and NO. Down-regulated NF-κB (p65) in nucleus	[21]
*Sargassum hemiphyllum* Ext: W, 100 °C, 30 min C, FD 95% E pptn., C, FD	Aarachidonic acid-induced ear inflammatory in mice Decreased ear swelling and erythema. Decreased production of myeloperoxidase, NO, IL-1β, IL-6, and TNF-α. Reduced area of neutrophilic infiltration in ears	[193]
*Sargassum muticum* Ext.: W, heating up to 170 °C Alginate pptn Fuc:Gal + Xyl + Man:Glu1:0.94:0.24	Reduced IL-6 production stimulated by IL-1β. Up-regulated Nrf-2 levels and the expression of its transcriptional target genes HO-1 and SOD-2. No attenuation of chondrocyte senescence	[104]
*Sargassum patens * Ext: crude enzyme from *Shewanella oneidensis* PKA 1008, LRS: 50; Seaweed:crude enzyme ratio 1:1(*v*/*v*), 30°C, 48 h	LPS-induced RAW 264.7 cells Inhibited secretion of IL-6, IL-1β, and TNF-α cytokines	[28]
*Sargassum polycystum* Pre-Ext: 95% E; 10% FMD in 95% E, 8 h, 37 °C, E washing Ext: Celluclast (0.5% E/S), pH 4.5, 50 °C, 24 h. Alcalase (0.5% E/S), pH 8.0, 50 °C, 24 h Neutraliz., 5 M CaCl_2_ alginate pptn, concent., 95% E pptn 75% E washing; C 62.9% carbohyd.; 27.5% sulf; 0.1% prot; 3.4% polyp., 0.5% ash	RAW cells Reduce the NO levels related to the reduced production or activity of iNOS. Down-regulated PGE2, reduced COX-2, TNF-α, IL1β, and IL6 levels	[187]
*Sargassum siliquosum * Pre-Ext: (HTHPP), 95% E, RT, 4 h Ext: MAE: W, LSR 15; MAE, 750 W, 10 min, F; Dl; 95% E pptn; IE pptn; C, Dl; 2% CaCl_2_ alginate pptn Purif: AEC; Dl, F, FD Depol: 0.1 M H_2_O_2_, 60 °C, 60 min 64.5% carbohyd., 19.5% sulf., 6.1% uronic ac., 9.9% phen. + prot., 31.5 and 3.2 kDa	LPS-stimulated RAW264.7 macrophage cells Suppressed TNF-α production	[113]
*Sargassum swartzii* Pr-Ext: 95% E, 3 stages; 10% FMD in 95% E Ext: Celluclast (0.5%), pH 4.5, 50 °C, 24 h; Filtr, Alcalase, pH 8.0, 50 °C, 24 h; neutr.; CaCl_2_ alginate pptn; neutraliz.; conc. FD, Pur: IEC 61% carbohyd., 34% sulf.; 0.4 % prot.; 0.3% polyphenol Fuc:Gal: Glu:Others ratio 82.5:3.2:1.3:13.0	LPS-stimulated RAW 264.7 macrophages Decreased NO production, acted on mediators such as iNOS, COX-2, and pro-inflammatory cytokines (TNF-α, IL-6, and IL-1β) Suppressed TLR-mediated MyD88, IKK complex, ultimately hindering NF-κB and MAPK activation	[137]
*Sargasum thunbergii* Ext: hot-water extraction, 0.5 M NaOH, 4 °C, 10 h; Amylase, pH 2.0, 37 °C, 2 h; conc.; E pptn, D; Deproteinization Purif: AEC; D, FD; 98.9% carbohyd. Fuc:Gal (mol) 1.2:1, 373 kDa	LPS-stimulated RAW 264.7 mouse macrophage cells Reduced TNF-α, IL-6, and COX-2 mRNA expression	[194]
*Sargassum weizhouense* Pre-Ext: 95% E, 80 °C, 8 h Ext: 1% papain, 50 °C, 1.5 h; filtr; conc., 95% E, 100% E	PCV2 infection Inhibited histone acetylation and the production of inflammatory cytokines, improving the resistance of the host	[195]
*Turbinaria ornata* Pre-Ext: 95% E; 90% E, 10% FMD, 8 h Celluclast 0.5%, pH 4.5, 50 °C, 24 h Alcalase 0.5% E/S, pH 8.0, <50 °C, 24 h CaCl_2_ alginate pptn; neutraliz; 95% E pptn AEC, 60.3% carboh., 38.3% sulf., 0.24% prot., 0.25% polyph	LPS-treated RAW 264.7 macrophages Inhibited NO production. Down-regulated expression of iNOS and COX-2 Zebra fish embryos Reduced the NO, ROS, and cell death levels; down-regulated inflammatory mediators, iNOS, and COX-2	[136]
*Undaria pinnatifida* Comercial fucoidans hydrolyzed CuAc pH 7.5, 60 °C, 9% H_2_O_2_, 5 h Cu removal, neutralization, Dl (1 kDa), FD LMWF (5–30 kDa)	LPS RAW264.7 cells Regulated signaling pathways, attenuated IL-1β, IL-1, and TNF-α, and the degradation of phosphorylated p38 MAPK, ERK1/2 and JNK. Blocked NO and ROS. Inhibited iNOS and COX-2	[24]
*Turbinaria decurrens* Pre-Ext.: 85% E, RT, 12 h W, 65 °C, 1 h C, 1% CaCl_2_ alginate pptn; 99% E sequential pptn Pur: dW heated with 3.0 M HCl, 3 h, cooled, C, neutr., E pptn, W washing, FD 54.8% polys., 23.5% sulf, 3.4 % uronic, 2.7% prot. 9.3% Fuc, 12.6% Gal, 9.6% Man, 6.4% Rha, 11.4% Xyl	Reduced LPS-induced cytotoxicity in IC-21 macrophages Formalin-induced paw edema in mouse model Decreased the MDA and increased SOD, CAT, GPx, GST, and GSH activity. Retained p65/NF-κB transcription factor. Down-regulated expression of pro-inflammatory mediators such as IL-1β, COX-2, and MMP-9	[196]
*Undaria pinnatifida* Ext: Triton solubilized; hot, acidic water or hot alkaline water Depol: W, copper acetate monohydrate, 9% (*v*/*v*) H_2_O_2_, 60 °C, 5 h, Cu removal, neutr., diafiltration (1 kDa), FD AEC (DEAE), conc., Dl, LMWF, 1 kDa	LPS-stimulated spleen cells Suppressed the production of IFN-γ Collagen-induced arthritis mice model; spleen cells Inhibited Th1-mediated responses, reduced collagen-specific IgG2a levels in serum	[115]
*Undaria pinnatifida* Pre-Ext: 85% EtOH, 70 °C, 2 h Ext: HCl (pH 2), RT, 24 h. 582.5 kDa or US, 80% amplitude, 6 h, HCl (pH 2) neutr., Dl (3.5 kDa), FD, 390.6 kDa	LPS-induced inflammation in Raw 264.7 cells Suppressed iNOS and COX-2, and JNK1/2 and p38 phosphorylation	[147]
*Undaria pinnatifida* Ext: MAE, W, 160 °C Alginate pptn Fuc:Gal + Xyl + Man:Glu 1:0.93:0.24	Reduced IL-6 production stimulated by IL-1β. Up-regulated Nrf-2 levels and the expression of its transcriptional target genes HO-1 and SOD-2. No attenuation of chondrocyte senescence	[104]

A: acetone; E: ethanol; M: methanol; MC methylene chloride; FMD: formaldehyde. HTHPP: high temperature and high-pressure puffing; MAE: microwave-assisted extraction; RT: resin treatment; SWE: subcritical water extraction; W: water extraction. C: centrifugation; D: drying; Dl: Dialysis; FD: Freeze-drying. COX-2: cyclooxygenase-2; IL-1β: interleukin-1 beta; IL-6: interleukin-6; iNOS: inducible nitric oxide synthase; NF-κB: nuclear factor kappa B; PGE2: prostaglandin-E2; TNF-α: tumor necrosis factor-alpha; NO: nitric oxide; JNK: Jun N-terminal kinase; NF-κB: nuclear factor kappa B; MAPK: mitogen-activated protein kinase. AEC: anion exchange chromatography; CuAC: copper acetate monohydrate.
